# Promoting a culture of sharing the error: A qualitative study in resident physicians' process of coping and learning through self-disclosure after medical error

**DOI:** 10.3389/fmed.2022.960418

**Published:** 2022-10-21

**Authors:** Mari Asakawa, Rintaro Imafuku, Chihiro Kawakami, Kaho Hayakawa, Yasuyuki Suzuki, Takuya Saiki

**Affiliations:** ^1^Medical Education Development Center, Graduate School of Medicine, Gifu University, Gifu, Japan; ^2^Department of General Internal Medicine, Sakai Medical Center, Osaka, Japan

**Keywords:** medical error, emotional distress, self-disclosure, resident physician, high reliability organizations (HROs)

## Abstract

**Purpose:**

Most physicians, including residents, experience significant emotional distress after making medical 11 errors. As high reliability organizations (HROs), hospitals must not only support physicians' emotional recovery but also promote their learning from errors. Self-disclosure is a process of communication in which individuals reveal information about themselves to others. While many previous studies have focused on investigating the effectiveness of self-disclosure, little is known about the process itself. Therefore, this study aims to explore residents' processes of coping with their emotional distress and learning through self-disclosure after making errors.

**Methods:**

Semi-structured interviews were conducted with 22 residents in their second year from two Japanese hospitals where informal error conferences guided by senior residents are implemented regularly. In the interview, four core questions were posed regarding the nature of the error/incident, their emotions and behavior after the error, ways of self-disclosure, and the results of error-sharing in the conference. Interview data were thematically analyzed, drawing upon disclosure decision model as the theoretical framework.

**Results:**

Five phases emerged from the analysis: (1) emotional distress and reactions before self-disclosure; (2) self-disclosure to individuals to achieve social rewards; (3) emotional sublimation after self-disclosure to individuals; (4) sharing errors in groups for learning opportunities; and (5) transforming the perspectives on overcoming and learning from errors.

**Conclusion:**

This is the study to demonstrate that various types of self-disclosure were embedded in the processes of residents' recovery and learning from medical errors. The study suggests that a better understanding of the processes of residents' coping with their distress and learning from their errors through self-disclosure is fundamental to the creation of a “culture of sharing errors” in hospitals as HROs.

## Introduction

Most physicians, including residents, experience significant emotional distress after making medical errors or experiencing adverse events (1–4). When an adverse event occurs, it can be a traumatic experience not only for the patient and the patient's family but also for the physicians involved (5, 6). Physicians often experience significant emotional distress in such situations such as shame, guilt, self-doubt, depression, and even suicidal ideation (7–10).

Of these negative emotions, shame and guilt are known to be powerful and ubiquitous in response to negative events. Shame is regarded as negative feeling focusing on the “self” and is associated with negative evaluations of the entire self as “small” and “worthless.” Guilt, by contrast, is regarded as a positive feeling focusing on “behavior,” which is associated with repairing action (11, 12).

Residents are a particularly important and vulnerable population needing support in cases leading to such emotions as their early experiences of medical errors may shape their future behavior and coping skills (13). The influence of these emotions on healthcare providers has been discussed in medical literature, and further discussion of the impact on residents is needed. Clinical teachers should cultivate engaged, empathetic, and shame-resilient learners using non-humiliating, action-based, and empathetic approaches (12). From that point, it is essential for clinical teachers and concerned individuals to discuss and develop strategies to support residents after making medical errors. Therefore, this study focused on residents' responses to medical errors.

From an organizational perspective, hospitals have a dual role of transforming medical care as high-reliability organizations (HROs), defined as organizations that must be “nearly error-free” to avoid catastrophes despite a high level of risk and complexity (14, 15). First, hospitals as HROs must build a system to learn from medical errors such as reflective error conferences. Second, it is crucial that organizations provide emotional support for physicians who experience significant emotional distress to avoid losing valuable human resources.

Previous studies have focused on investigating physicians' cognitive and emotional processes after medical errors. Scott (8) identified six stages of the natural history for the healthcare provider after adverse patient event: (1) chaos and accident response; (2) intrusive reflections; (3) restoring personal integrity; (4) enduring inquisition; (5) obtaining emotional first aid; and (6) moving on to dropping out, surviving, and thriving. In line with this process, several studies have reported that an organizational support program is needed, such as supervisor support, a private counseling system, team meetings, formal conferences (such as Morbidity and Mortality conferences), and an organizational constructive support system (e.g., Scott's three-tiered system and medically induced trauma support services) (8, 16). Studies have shown that an organizational support program that provide trained peer support for health care providers, mainly nurses, has reduced turnover among them (17).

From an individual perspective it has also been noted in recent decades that self-disclosure to colleagues or peers provides both emotional support and learning opportunities for further growth (13, 18). Plews-Ogan noted that physicians wanted “a peer and an ear” to openly discuss their errors with a supportive colleague. Self-disclosure is a process of communication in which individuals reveal information about themselves to others. The information can be descriptive or evaluative and can include thoughts, feelings, aspirations, goals, failures, successes, fears, dreams, likes, dislikes, and preferences (19). Although previous studies have shown that self-disclosure to colleagues is important, less is known about the process of self-disclosure: when, to whom, and for what purpose physicians disclose, including factors that prevent them from disclosing.

Furthermore, although an informal error conference might be useful, as is a formal one (13, 20), it remains to be determined when and with what purpose physicians intend to present informal conferences and what factors facilitate or discourage them. Engel encouraged more widespread error conferences, including “informal” along with “formal” discussions. Since individuals have varying preferences for these different forms of error disclosure and discussion, combining “formal” and “informal” discussions may generate powerful synergy (13). Research has shown that engaging residents in blame-free discussions encourages communication about learning from errors (21), and, from an organizational perspective, no-blame practices have been found to be beneficial in HRO environments where learning and reliability issues are particularly relevant (22).

Considering these studies, it is conceivable that residents who have made medical errors are expected to cope with their emotional distress by disclosing errors to their colleagues personally as well as learning through sharing their experiences in informal conferences. However, no study has fully explored the complex process of self-disclosure from the residents' perspective.

Disclosure Decision Model (DDM) is a framework that can be useful in exploring this issue. DDM specifies a cognitive process resulting in decisions that affect the content, depth, breadth, and duration of self-disclosure (23). In the first stage of DDM, self-disclosure is selected as the primary strategy to achieve one of five social rewards: social approval, intimacy, relief of distress, social control, and identity clarification. The second stage involves decisions on whether disclosure is an appropriate strategy and who is an appropriate target. In the third stage, decisions are made regarding what to disclose: how broadly, how much, and how intimately. Based on individuals' perceptions of the situation and target, subjective utility and subjective risk are assessed. Ultimately, this subjective assessment determines the three main dimensions of self-disclosure (breadth, duration, and depth of self-disclosure). Breadth refers to the number of topics covered by disclosure. The duration of disclosure is the sheer amount or persistence of the disclosure. Depth is defined as the intimacy level of disclosure, described as a combination of intense emotional and potentially negative disclosures (23).

Using DDM, we developed the following research questions:

- What is the process of coping with emotional distress through self-disclosure from the residents' perspective?
- How do residents develop an attitude of learning from errors through the process of self-disclosure?
- What factors accelerate or discourage self-disclosure in informal conferences?

This study aims to provide deep insight into how surrounding supporters and clinical teachers should support residents who make errors and how informal error conferences should be conducted.

## Materials and methods

### Qualitative research design

This study adopted a qualitative research methodology informed by the interpretivist paradigm to conduct an in-depth analysis of the complex phenomenon of residents' perspectives on emotional distress and learning from errors through self-disclosure after making errors. Qualitative data were collected through 22 audio-recorded face-to-face, semi-structured, in-depth, and open-ended interviews. All data were analyzed inductively. To identify cognitive and emotional processes after making errors from the perspective of self-disclosure, we chose reflexive thematic analysis to elicit subjective meanings, which involved generative coding and theoretical interpretations by the research team.

### Setting and participants

We conducted the study among residents in their second year at two Japanese postgraduate training hospitals where informal error conferences were conducted. Potential participants were all residents in their 2nd year at these hospitals, with sufficient clinical experience and experience of episodes of errors. Hospital A had 13 and Hospital B had 5 residents in their 2nd year. The programs at the two hospitals were based on a rotation system in each department, and there was no bias in the residents' majors. The conference was implemented regularly (once per week) and was guided by senior residents. Both hospitals had formal reporting systems for medical errors (including incidents) for patient safety. Medical errors were discussed in the medical safety committee and explained to patients and their families based on the conclusions of the committee. Both hospitals also had Morbidity and Mortality conferences. In addition, both had their own mentorship programs to support each resident throughout their residency.

We recruited participants by asking for volunteers from the two hospitals. We intentionally included residents who had experienced an incident or medical error and had attended an informal error conference. Following approval from the research ethics board, over a 2-year period, 17 residents from Hospital A and 5 residents from Hospital B were recruited (13 male, 9 female). All of these residents agreed to participate in the study, and no one refused. The sample size was estimated to be sufficient based on the principle of theoretical saturation (22). The participant details are presented in Table 1. The mean age of the participants was 27.2 years old.

**Table 1:** Participants.

**Code**	**Gender**	**Hospital**	**Code**	**Gender**	**Hospital**
1	F	A	12	F	A
2	F	A	13	M	A
3	M	A	14	F	A
4	M	A	15	M	B
5	F	A	16	M	B
6	M	B	17	M	A
7	M	B	18	M	A
8	M	B	19	M	A
9	M	A	20	F	A
10	M	A	21	F	A
11	F	A	22	F	A

### Patient and public involvement

No patients were involved.

### Data collection

Semi-structured interviews were individually conducted in Japanese by the first author (MA) in a private room with a safe environment. Interviews lasted approximately 40 min each. Before the interviews, definitions of the terms used in this study were explained to the participants. For example, following the Institute of Medicine, “medical error” was defined as “the failure of a planned action to be completed as intended or the use of a wrong plan to achieve an aim” (1). A “near-miss” was defined as “an event or situation that could have resulted in an accident, injury, or illness, but did not, either by chance or through timely intervention” (24). “Error” is understood as a word that encompasses a wide range of mistakes from the near-miss to a serious adverse event to cast the widest possible net (13).

Based on the interview guide (Table 2), we explored six core questions regarding the content of the error that had a deep impact on the participants, their emotions and behaviors after the event, the type of self-disclosure (when, to whom, and for what), their emotional changes after self-disclosure to individuals, why they shared (or did not share) the error in the informal conference, and their emotional changes after the conference. The interview data were digitally recorded and transcribed verbatim.

**Table 2:** Interview guide for all participants.

1. Have you ever had any clinical error or incident that impacted you in the past? Please describe it as far as it's all right with you.
1) How long ago did the event occur? 2) What type of event was it? (error or incident) 3) Describe your specific role in the event. 4) Describe the patient outcome.
2. How did you feel immediately after the event?
3. How did you handle those feelings?
4. Did you talk to anyone about the event?
1) Who did you talk to? 2) Describe the situation in which you talked to him/her. 3) Did you talk about your feeling to him/her? 4) Is there any change of emotion or recognition regarding the event after talking to others?
5. Did you share this event in the informal conference?
1) When did you share it? 2) Why did you decide to share the event in the conference (or not)? 3) Are there any changes of emotion and recognition regarding the event after sharing it in the conference?
6. What helped you to recover from your emotional distress regarding the event?
7. What do you think are the better ways for dealing with your emotional distress?
8. Based on your experience, what would you do to support your colleagues who make a medical error?
9. What kind of supports do you think are needed for residents who make a medical error?
10. Have you ever learnt how to deal with medical errors in your professional training?

This study was approved by the Institutional Review Board of the Gifu University Graduate School of Medicine as the overarching ethical committee, after the approval of the Ethics Committees of the two hospitals.

### Data processing

The interviews were audio-recorded, and verbatim transcripts were produced from the recordings. The first author translated Japanese transcripts into English. During this process, private identifiers were replaced with anonymized data. Coding software (NVivo V.12, QSR International, Massachusetts, USA) was used to manage and organize the data. To report this research in an audit trail, this study also carefully documented all components of the data analysis process, including raw data, coded transcripts, researchers' notes, and analysis products.

### Data analysis

This study employed Braun and Clark's reflexive thematic analysis in an inductive manner (25). Following the six-phase process of thematic analysis developed by Braun and Clarke, all researchers systematically reviewed the transcribed data to better understand its content. This is called the familiarization phase. The second phase is coding, in which the text data are broken down into small units according to their beliefs, actions, events, or ideas. In this phase, MA and TS individually performed the initial coding of data from the participants. The third phase generates the initial themes. In this phase, all members compared the results of individual initial coding and identified broader significant patterns of meaning (i.e., theme). On this basis, MA coded the remaining transcribed data. Specifically, each small unit was coded with an interpretive description and grouped into more abstract themes from the perspective of self-disclosure.

At this stage, a disclosure decision model by Omarzu (23) was employed as an analytical framework to further explore the cognitive process resulting in decisions that affect the content, depth, breadth, and duration of self-disclosure. The fourth phase was reviewing themes, where all researchers reviewed the initial themes developed in the previous phases iteratively to ensure that the researchers' interpretations were congruent with the presented data. The researchers then defined the final themes in the fifth phase, which involved developing a detailed analysis, identifying the focus, and determining the story of each theme. Finally, in the sixth phase of writing, the researchers contextualized the analysis in relation to the existing literature. Standards for Reporting Qualitative Research (SRQR) were used to write the report (26).

### Trustworthiness of data analysis

To enhance the trustworthiness of the qualitative analysis, two researchers (MA and TS) were independently involved in coding and categorizing the data. The authors then crosschecked their data interpretation and analysis. The preliminary findings were carefully reviewed multiple times by all members of the research team to establish the validity of the data analysis. We also conducted a member check in which some available participants were asked to evaluate the researchers' interpretation of the data.

## Results

Five phases and 11 themes emerged from the analysis, presented in Table 3. In the following sections, residents' experiences and perceptions in each phase are described in detail.

**Table 3:** Summary of the result emerged from the analysis.

Phase 1—Emotional distress and reactions before self-disclosure
Theme 1-1: Chaotic emotional distress after medical errors Theme 1-2: Contrastive emotional reactions to shame and guilt
Phase 2—Self-disclosure to individuals to achieve social rewards
Theme 2-1: Self-disclosure to peers for relief of distress Theme 2-2: Self-disclosure to senior residents for relief empathy and non-clinical advice Theme 2-3: Limited self-disclosure to attending doctors and formal mentors Theme 2-4: Non-self-disclosure to anyone else
Phase 3—Transition from healing phase into learning phase
Theme 3-1: Emotional sublimation by self-disclosure
Phase 4—Sharing errors in groups for learning opportunities
Theme 4-1: Conflicting attitude toward sharing errors in groups caused by situational factors Theme 4-2: Double-edged sword—self-efficacy after sharing in groups
Phase 5—Transforming the perspectives on overcoming and learning from errors
Theme 5-1: Recognizing the importance of sharing errors in groups Theme 5-2: Motivation to develop “a culture of sharing errors”

### Phase 1—Emotional distress and reactions before self-disclosure

In this phase, immediately after committing medical errors, residents' thoughts were dominated by panic and depression followed by shame and guilt. At that time, there was no room for disclosure to others.

#### Theme 1-1: Chaotic emotional distress after medical errors

Immediately after the errors, the residents were terribly upset and tried to survive a critical situation while experiencing panic. It was not until the situation stabilized that they gradually began to experience depression and loss of confidence. A more serious error (e.g., an adverse event that affected a patient) was associated with stronger emotional distress. Even when the error was not critical and did not influence the patient's life, they were often shocked.

- I lost my confidence and faced the reality. It was like, “I didn't even know that” (1).

Indeed, although the emotional distress seriously influenced their work, they were not allowed to use it as an excuse to leave their work. For example, they felt it was difficult to focus on their work because their mind was full of distress. It was also stressful to continue to work without showing emotional distress to other patients while pretending to be okay.

- It was hard, and there was such a feeling in the corner of my heart, so it felt strange like that half of the desk of my mind was occupied (3).
- The hardest thing was that [I was] feeling depressed, but I was not allowed to show it to the other patients (13).

#### Theme 1-2: Contrastive emotional reactions to shame and guilt

When the residents tried to look back on their error experiences, they were gripped by shame and guilt. They felt more ashamed if their self-assessment was based on others' evaluations, comparing themselves with colleagues. Therefore, they were anxious about others looking down on them. These residents felt shame despite the relatively small number of errors. Additionally, some residents felt more shame because they thought they should be humble regardless of others' evaluations.

- My colleagues would see me like, “That guy screwed up.” I'm a person who worries about what others think, so I felt quite embarrassed (4).

In contrast, the residents had a sense of guilt when thinking about their influence on the patients and families rather than themselves. Poorer patient outcomes were associated with stronger guilt. Sometimes, even their own death could cross their mind as atonement due to the strong sense of guilt. Similarly, residents developed a sense of guilt toward their attending doctors who had taken over patient care after they made the error. Some residents felt shame focused on self-esteem followed by a sense of guilt focused on patients.

- I thought that I was an irresponsible person as I had put the patient's life in danger by inappropriate care. I felt that, if he/she had died, it would have been better if I had died instead (20).
- The fact that I messed up in front of my attending doctor made me feel twice as sorry about it (5).

### Phase 2—Self-disclosure to individuals to achieve social rewards

After reflecting on their own experiences, the residents started to seek individual targets for self-disclosure. This is because they recognized that they could not manage their emotions, although they tried to rebuild themselves by organizing their narratives. Accordingly, the desire to organize their emotions by talking with someone welled up subconsciously. Regarding self-disclosure to individuals, residents estimated the utility and risk of self-disclosure when choosing targets in advance.

#### Theme 2-1: Self-disclosure to peers for relief of distress

The residents disclosed narratives of errors to their peers not to seek any advice but rather to seek acceptance and sympathy without judgement. Therefore, they chose peers who shared similar circumstances, difficulties, and hardships in the same training program. Female residents, in particular, preferred female peers because they expected that female peers would give them sympathy without judgement or criticism. Even having someone just listen to and accept their stories could allow them to feel secure since verbalization helped them organize their thoughts and emotions.

- I may be very naive, but my peers don't blame me. I'm sure they understand how I feel (20).
- I [would] talk to one of my peers about it, and if I was depressed, he/she would listen to me as well. That's how it is with both of us (11).
- When I disclose to my female peers, they definitely say things that make me feel better. They empathize with me just by listening to me. I talk to them because it's comfortable (12).
- Just the fact that he/she listens to me is quite refreshing. If he/she can understand that I'm going through something like that, then I'm OK (9).

On the other hand, residents never confessed their errors to peers they considered rivals because the subjective risk of shame and frustration by comparing themselves with peers prevailed. Additionally, in the case of serious adverse events, they may not have talked to their peers while utility was low because they believed it was beyond their peers' capacity to accept.

- It was hard to talk to my peers about it because I saw my peers as rivals, or I was frustrated that I was not capable like them (5).
- I thought this was a bit too much (incident) for my peers to comfort me (1).

#### Theme 2-2: Self-disclosure to senior residents for empathy and non-clinical advice

After making errors, some residents self-disclosed to senior residents with whom they had a trusting relationship to obtain both empathy and non-clinical advice. They expected senior residents to encourage them without criticism and give them advice on how to overcome the hardship by sharing their own failure stories. Seniors' failure stories could reduce residents' anxiety and allow them to positively think that they can overcome their errors. They believed that experienced senior residents could accept even serious adverse events that were beyond their peers' capacity to accept.

- When I talked to my seniors who have overcome failures, I felt that I could grow in a positive way on my mindset for the future (5).
- What I expect to hear are failure stories of my seniors, such as, “I was like this when I was young.” I think that, deep down, I want to hear that such a great senior was like this when he or she was a junior (14).
- I think it's valuable to listen to failure stories of senior residents. I think it has a completely different weight. I believe that those hardships have made them who they are today (19).

However, when they did not have any senior residents they could trust close to them, they never disclosed to the senior residents because of the high risk of being blamed and disrespected.

- I can't tell my seniors my mistake. I have an obsession that I will be disrespected. They are all excellent doctors, and I don't think anyone would make such a mistake (12).

#### Theme 2-3: Limited self-disclosure to attending doctors and formal mentors

The residents considered that attending doctors and formal mentors were not the right people to disclose their errors to because of the high risk and low usefulness in terms of relief of emotional distress. They believed that attending doctors would not accept their emotions unconditionally, worried about their evaluation, and cared about the limited time available.

- My attending doctor is not my mother. I don't have any reason in which I feel free to talk with them (12).
- I don't want to be downgraded because some of the attending doctors are in a position to evaluate me (15).

Few residents intended to disclose to their attending doctors to ask for specific clinical advice, rather than sympathy or encouragement. In such cases, they confessed to the attending doctors only after organizing their emotions and carefully planning a reasonable story through disclosure to their peers or senior residents.

- It's not easy to talk to my attending doctor about my failure story like this. [If I would really like to talk,] first of all, I would talk with my peer who is at the same level as me and organize my thoughts. [...] After preparation with peers, I [would] talk to my attending doctor carefully (12).
- It's not something that I need to ask my attending doctor to take the time to do. They are always busy and not available. I have a lot of clinical matters I need to ask for advice right now, so I'd rather spend my time on those (18).

Formal mentors largely depend on each mentor's character and the mentor–mentee relationship. When the residents both had no good relationships and feared the negative consequences of self-image, they never chose their mentors as a target to disclose but rather peers and senior residents.

- My mentor was so strict that I could never tell him about my errors. I've never said it once (14).

#### Theme 2-4: Non-self-disclosure to anyone else

Residents did not disclose to anyone when they recognized that the utility of disclosure was low or the risk was too high. This strongly depended on their perception of self-disclosure. Some residents recognized the utility of self-disclosure as low; they kept their experiences only in their minds because they recognized that seeking emotional healing through self-disclosure would spoil them or that sharing emotions with others would not give them catharsis, and there would be no benefits.

- I don't need to talk to others about my errors because the answer “I was wrong” is already inside of me. I think I'm being too easy on myself to ask for encouragement (21).

However, some of them may have expressed that self-disclosure was not useful in hiding feelings of shame, pretending to be strong. One of the male residents had a gender stereotype that men should not disclose themselves easily.

- [I don't disclose to anyone because] I'm a man. I think women can talk and let out their emotions and refresh themselves, but I'm not that type of person (16).

Some residents recognized that the risk was too high; they did not disclose their error to anyone because they worried about how they would appear to others, considering self-disclosure as strongly shameful and distressing. Consequently, when they decided not to confess to anyone, they prepared to take it to their heart, carrying guilt for the rest of their lives without forgiving themselves.

- I still remember all my errors. I forget what I forget in my sleep. And what I don't forget, I should carry with me for the rest of my life (14).

### Phase 3—Transition from healing phase into learning phase

The residents were able to focus on learning from their errors and applying them to the future only after they had healed their emotions by reflecting on the past through personal self-disclosure.

#### Theme 3-1: Emotional sublimation by self-disclosure

Residents could relieve their emotional distress after they verbalized their narratives through self-disclosure. They also organized their emotions and perspectives, including shame and guilt. As a result, their thoughts shifted from the past to the future, making the next phase “To learn from the experience of the error and make use of it in the future.” They found that studying medicine much harder was not sufficient to rebuild their confidence or bring them to the next phase.

- [By talking about it,] I can organize it rationally and emotionally. I do self-reflection too, but when I talk to others, it means that I can summarize it within myself to some extent (8).
- Talking about it helped me move forward. I thought, “I can't help what I've done; I have to make the most of it next time.” When she said, “Making use of it for the next patient is the meaning of this mistake,” I felt my mind move forward, not to the past, but to the future (5).
- Of course I had studied much harder, but studying alone did not regain my confidence (1).

Although they still had feelings of shame, some of them tried to take the hardship to heart, using their feelings of shame to avoid repeating the same errors in the future. Even when they had a strong sense of guilt, they were able to forgive themselves through self-disclosure, reaching this mindset, and making use of the error experiences the next time.

- I thought it would be better if I died instead, but, in the end, I wouldn't die, and I would probably continue to be a doctor. I really felt sorry for this patient, but it was about 2 weeks later that I came to the conclusion that I had to make the most of this experience (20).

### Phase 4—Sharing errors in groups for learning opportunities

In Phase 2 (self-disclosure to individual targets), individual beliefs and relationships with others significantly influenced self-disclosure behavior. In Phase 4, in contrast to Phase 2, situational factors strongly influenced residents' self-disclosure behavior. Even though they had organized their emotions in Phase 3, they were always conflicted between the expectation of learning and fear of psychological distress with regard to sharing errors in groups.

#### Theme 4-1: Conflicting attitude toward sharing errors in groups caused by situational factors

In general, the majority of the residents positively regarded sharing errors in groups. Some residents who did not personally self-disclose shared their errors in groups at the last minute. While they were conflicted between the positive and the negative feelings, they believed that sharing errors was beneficial for learning for themselves and others. On the other hand, they had negative feelings that they could be blamed or criticized. Therefore, they sometimes did not share their errors with the groups.

- [I had talked about my error with my peers before...,] and I have been able to organize and accept my feelings that the patient in my case couldn't be saved, so I shared it on the conference (2).
- It might be a good case to learn from, but I wouldn't present a case [at the conference] where my practice was terrible. I'm sure I won't (14).

Whether residents self-disclosed was very situational. The residents had to overcome various conflicts such as cases, emotions, and atmosphere before they shared their errors in groups at the conference. First, they carefully selected cases to share. In particular, they believed that difficult cases had less risk of being criticized. They avoided sharing easy and simple errors because they believed that those cases would not be a lesson for others to learn.

- Difficult cases are easy to share [in the conferences]. I think it is because my lack of knowledge is not exposed. It's easy to present a case like, “He/She had a dissection of the celiac artery.” That's difficult for anyone. If I were to say, “He/She had an aortic dissection,” most of the participants would think, “So what?” (12).

Second, they were also influenced by their emotions. For instance, they avoided sharing simple errors that had the risk of causing shame by exposing their lack of ability. Even when their sense of guilt was strong, they encouraged themselves to share the errors to confront and overcome their terrible experiences.

- I felt a lot of guilt. I thought that “I don't want others to know my error” was just running away. I really thought, “I shouldn't run away from this case (20).

Third, they were strongly influenced by the atmosphere at the conference. When they witnessed others being blamed (or criticized) or noticed some participants who often criticized colleagues, they thought the risk of being blamed was too high; therefore, they did not share the cases that they had prepared to share.

- When I saw the other resident being criticized [in the conference], I think, “Oh no, is it OK to share my case? I don't want to do.” The atmosphere made me not want to share it (11).

#### Theme 4-2: A double-edged sword—self-efficacy after sharing in groups

Although the residents managed their conflicted feelings and shared at the conference, there was a fine line between success and failure. Some residents gained self-efficacy when they recognized that it resulted in not only their own learning but also that of colleagues. Even when guilt was overwhelming, they eventually felt a sense of accomplishment like “overcoming the error” through sharing the error.

- I felt triumphant like “I'm glad I shared it.” I told them about my error, but I thought, “This is no shame” more than that (1).
- I thought it was helpful to everyone, and I also had a sense of accomplishment when I talked about my error (15).

In contrast, when the residents felt blamed (or criticized) despite motivating themselves to share, they were discouraged, demotivated, and not positive about learning from errors. When they experienced blame in the conference in the early days, they would not like to share their errors because of psychological trauma.

- [When I was blamed at the conference,] I just felt so bad. But it was due to my lack of study, so I was still depressed (12).
- [Being blamed] is too memorable. That hurt me so much in the early days that I don't want to share my cases after that (14).

### Phase 5—Transforming perspectives on overcoming and learning from errors

The residents overcame this difficulty and formed a positive perspective toward learning from their errors as they repeatedly had difficulty sharing in groups through various trials and errors. Additionally, some of them realized that the acknowledgment and nonjudgmental manner allowed them to develop a culture of sharing errors.

#### Theme 5-1: Recognizing the importance of sharing errors in groups

As they gained experience by sharing their own errors and listening to those of others, the residents began to realize the usefulness of sharing errors as well as the importance of mentally supporting each other.

They found the usefulness of error conferences in obtaining learning experiences by simulating in their minds without making errors. They developed gratitude toward their peers who shared errors. One of them noticed that sharing errors allowed the peers to realize that they were not the only ones who made errors, which reduced their sense of shame and created a virtuous cycle that encouraged sharing errors. As they accumulated experience as doctors, they felt more comfortable, which helped them notice the usefulness of sharing errors in groups.

- I'd like to thank my colleagues for exposing their own errors because we all can learn from them without making errors (1).
- I have a limited number of cases I can experience on my own. I think that knowing about others' cases is the same as having my own experience, which increases my experience (18).
- I came to realize that everyone has made errors too. I found that I'm not the only one. By me sharing my errors, my peers don't need to feel embarrassed, and it can also save patients. So the benefits are greater than the risks (19).
- I felt more comfortable, and I also got used to working as a doctor. The room in my mind reduced the disadvantages of sharing errors (19).

#### Theme 5-2: Motivation to develop “a culture of sharing errors”

As they gained more experience in clinical practice and error conferences, the residents gradually developed their own perspectives on sharing errors in the workplace. They became aware of the importance of error sharing in a safe environment. Some realized that they needed to be good listeners to create a culture of sharing errors.

- I don't think it's reasonable to blame him for it when someone shares an error. Since he knows that he failed, there is no need to say that anymore (18).
- Many doctors take things seriously, so I think that many of them tend to deny themselves when they make errors. We all need someone who acknowledges, like, “You're not a bad person, you're working hard enough.” And gives us a positive attitude to make use of errors next time (5).

For instance, some thought it was important to accept others' failure stories without blaming or criticizing and discussing them in a positive attitude to make it useful in the future. One of them also believed that exposing his own errors in groups would reduce the emotional distress of his peers and juniors, promoting a culture of error sharing.

- I want to tell my juniors, “We made these kinds of errors when we were in our first year.” There are probably many residents who feel depressed after errors and want to quit being a doctor. So, if I could tell them, “That happened to me too,” they would feel completely different (19).

## Discussion

### Summary

Five phases and 11 themes emerged related to the processes faced by the residents in this analysis. To the best of our knowledge, this is the first study to demonstrate the various types of self-disclosure embedded in the processes of residents' recovery and learning from errors.

**Figure 1:**
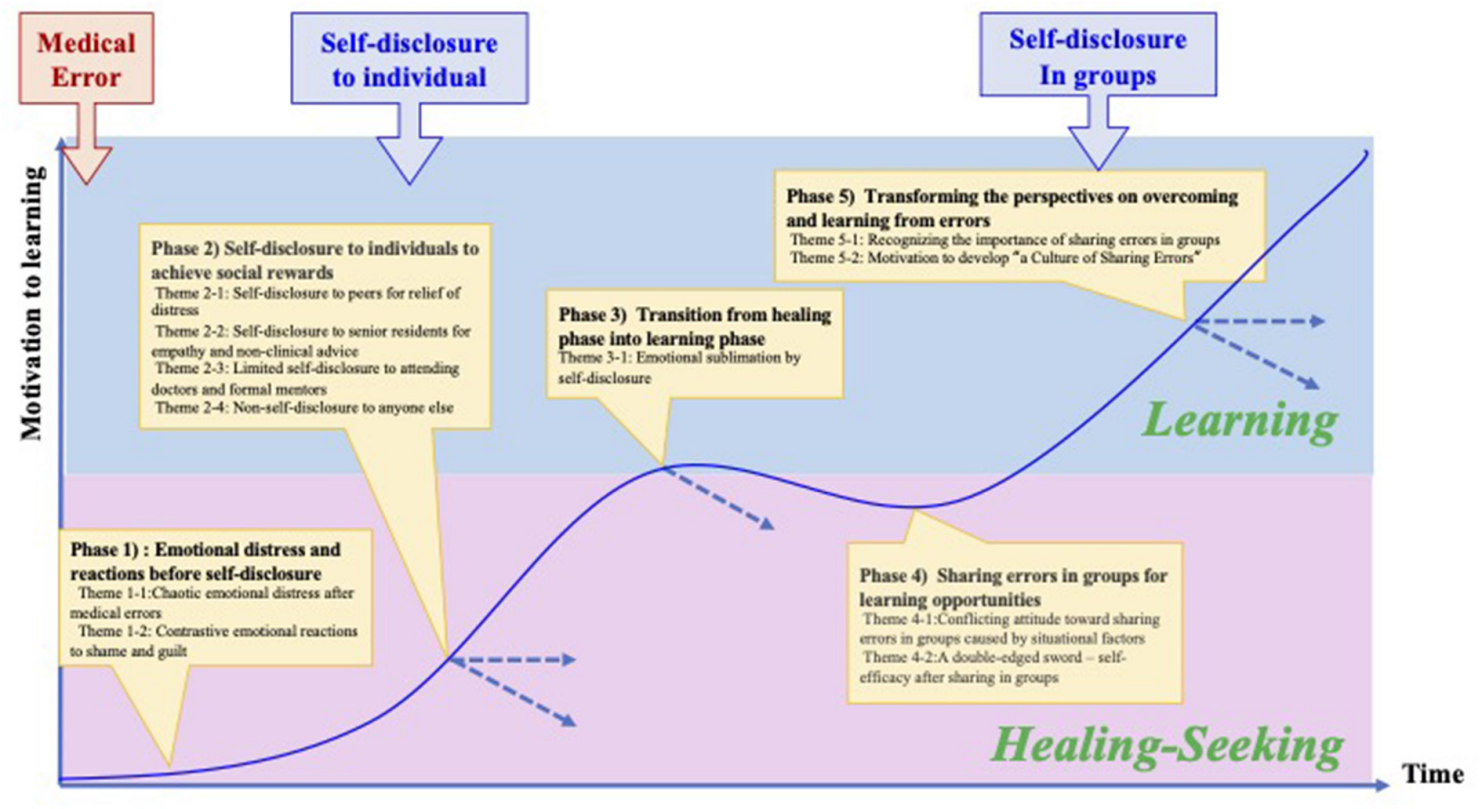
The shifting processes of residents' coping process, revealed through self-disclosure.

This study adds to the existing knowledge of the general trajectory of healthcare providers after adverse events and organizational support. Scott (8) sheds light on residents' in-depth emotional and behavioral patterns from the viewpoint of self-disclosure. Specifically, this study demonstrated the shifting processes of residents' coping through self-disclosure from healing-seeking behaviors through personal self-disclosure to learning behaviors in a conference that reinforces their self-efficacy (Figure 1).

Immediately after the errors, the residents were not motivated to learn from them (Phase 1). Only after self-disclosure to close colleagues to relieve emotional distress (Phase 2) did they develop motivation to share their errors in groups for their own and others' learning (Phases 3 and 4). Through experiences of self-disclosure in the group, they recognized the importance of “a culture of shared error” (Phase 5).

#### Catharsis and reciprocal effects

This study found two major roles for self-disclosure in the recovery process: catharsis and reciprocal effects. Previous studies have shown that self-disclosure to colleagues is effective in reducing emotional distress after making medical errors (13, 17).

The catharsis effect is the therapeutic release of tension and negative emotions as a result of self-disclosure (27). The results suggest that residents simply sought catharsis rather than clinical advice by verbalizing their emotions. We also found that the residents could move to the learning phase only after feeling “catharsis” through self-disclosure to their close peers. For example, if a Mortality and Morbidity conference is held at a time when one has not yet passed through the cathartic phase, there is a risk that one will not only be reluctant to learn but will also become more depressed.

In addition, the reciprocal effect is defined as the effect by which one person's self-disclosure elicits another person's self-disclosure (28). The results of this study demonstrated that the residents were encouraged to strengthen their relationships with colleagues through reciprocal effects from their peers and senior residents. Especially in self-disclosure to senior residents and attending doctors, most residents were more interested in others' failure stories than in clinical advice. The ability to talk about past failure stories depends on one's pride as a physician and whether there is an atmosphere in the workplace where one can talk openly about errors (29). If senior residents and attending doctors keep a stock of all kinds of failure stories in their minds, they can talk to the residents about their failure stories at the right time. Moreover, the results indicate that residents' self-disclosure has a high impact on encouraging sharing errors by colleagues. This could lead not only to great learning opportunities for residents but also to encouragement of sharing errors in workplaces.

#### Even incidents and a sense of guilt

Although it is well known that residents suffer various emotional distresses immediately after errors (6, 8, 9, 30), our study found that some of the residents had a sense of guilt even from incidents or near-miss events. Since these events rarely come to light despite large numbers, it is essential to recognize incident-level events to support residents affected by emotional distress as well as to promote medical safety (9, 13, 31). It was also found that an excessive sense of guilt after making an error should be considered. Previous psychological studies have explained that the sense of guilt is basically regarded as a positive feeling associated with recovery and empathy toward others (11). However, as noted in previous studies (12), our findings suggest that residents' excessive sense of guilt after an error can be associated with significant psychological distress.

#### Factors of self-disclosure in personal and “failure friend”

Based on the results of our study, it is equally important to have a “failure friend” (32), who is willing to self-disclose errors on a regular basis, rather than a counselor who is an expert but has no relationship with the individual. A “failure friend” can be explained as an empathetic work friend who understands the context—someone who is your safety net and for whom you can be a safety net in return. In this study, we revealed that residents' self-disclosing behavior was heavily based on the utility and risk concepts in the disclosure decision model (23). Therefore, it is not surprising that residents would choose intimate peers or senior residents as their first target rather than attending doctors or mentors. Although previous studies have suggested the establishment of a system of assigning psychiatrists and counselors to reduce emotional distress and prevent burnout and depression (8, 16), it is suggested that having a failure friend is useful for residents to learn from errors through catharsis.

#### High utility and high risk

Based on the disclosure decision model (23), the combination of high utility and high risk is associated with anxiety and distress, leading to sharing errors in groups. Therefore, the residents were conflicted between positive and negative attitudes toward it. If they decide not to share information, they will miss the opportunity to learn and grow as doctors. Even if they are present, they are traumatized when they encounter the experience of being blamed for errors in front of the audience. This is a double-edged sword that can lead to the formation of a long-lasting negative attitude toward sharing errors in groups.

#### Organizational culture of talking about errors

It should be noted that, through repeated self-disclosure of errors both individually and in groups and exposure to many situations in which peers disclosed their errors, the residents understood the importance of sharing errors, which in turn would lead to the development of a “hospital culture of learning from errors.” This understanding is essential for HROs to promote such a hospital culture. This is because such a culture, apart from a formal reporting system, forms informal norms that promote continuous organizational improvement and open dialogue beyond formal error-reporting systems (6, 33).

### Implications for the development of a culture of error-sharing errors

The following is a summary of the skills expected of colleagues and educators. It is expected that all staff will understand the importance of this culture and acquire these skills through faculty development and leadership training within the hospital.

- Just listen to residents' failure stories when confessed by them.
- Have a conscious attitude toward talking about residents' errors.
- Consider the phase of residents' healing and learning after errors, especially when deciding to hold a Morbidity and Mortality conference.
- Pay attention to residents' sense of guilt and encourage their self-forgiveness.
- Be “chosen” or “a person who elicit one's self-disclosure (openers)” (34).
- Create organizational opportunities to learn from sharing errors.

### Limitations

This study had several limitations. First, it was confined to interviews with residents of acute-care hospitals that held informal error conferences. Hospitals that hold informal error conferences may face fewer barriers for residents to share their errors than hospitals that do not. However, it is important to recognize that, even in such an environment, residents still experience difficulties in sharing errors. Second, this study focused on Japanese residents. The emotional distress experienced by residents after making errors and the conflict they felt when sharing errors in groups may differ from those in other cultures. However, as the global healthcare environment progresses, there seems to be a certain significance in understanding the characteristics of diverse cultures and in sharing the expertise of each country. Third, one of two hospitals had a smaller number of residents working there, resulting in a smaller sample size. Finally, it remains to be determined how much these implications for residents who have made errors has impacted both hospital culture of learning from errors and their future careers.

## Conclusion

This is the study to demonstrate that a variety of types of self-disclosure are embedded in the processes of residents' recovery and learning from errors/incidents. This study also revealed residents' in-depth emotional and behavioral patterns from the viewpoint of self-disclosure. The findings of this study may provide valuable insights to encourage residents to learn from errors as well as to develop an organizational attitude to learn from errors.

## Data availability statement

The raw data supporting the conclusions of this article will be made available by the authors, without undue reservation.

## Ethics statement

The studies involving human participants were reviewed and approved by the Institutional Review Board of the Gifu University Graduate School of Medicine. The patients/participants provided their written informed consent to participate in this study.

## Author contributions

MA and TS contributed to the conception, design, acquisition of data, analysis, interpretation of data, and drafting of the manuscript. RI, KH, CK, and YS made substantial contributions to the design of the study, data analysis, interpretation, and drafting the manuscript critically for important intellectual content. All authors reviewed, edited, and accepted the final version of the manuscript. All authors approved the final manuscript and agreed to be accountable for all aspects of the work.

## Conflict of interest

The authors declare that the research was conducted in the absence of any commercial or financial relationships that could be construed as a potential conflict of interest.

## Publisher's note

All claims expressed in this article are solely those of the authors and do not necessarily represent those of their affiliated organizations, or those of the publisher, the editors and the reviewers. Any product that may be evaluated in this article, or claim that may be made by its manufacturer, is not guaranteed or endorsed by the publisher.

## References

[1] Kohn LT Corrigan JM Donaldson MS. Errors in health care: a leading cause of death and injury. In: Kohn LT Corrigan JM Donaldson MS, editors. To Err Is Human: Building a Safer Health System. Washington, DC: National Academies Press (2000). 25077248

[2] Wachter RM. The end of the beginning: patient safety 5 years after “to err is human”: amid signs of progress, there is still a long way to go. Health Aff. (2004) 23 Suppl 1:W4–534. 10.1377/hlthaff.W4.534 15572380

[3] Newman MC. The emotional impact of mistakes on family physicians. Arch Fam Med. (1996) 5:71–5. 10.1001/archfami.5.2.71 8601210

[4] Delbanco T Bell SK. Guilty, afraid, and alone—struggling with medical error. N Engl J Med. (2007) 357:1682–3. 10.1056/NEJMp078104 17960011

[5] Kaldjian LC Jones EW Wu BJ Forman-Hoffman VL Levi BJ Rosenthal GE. Reporting medical errors to improve patient safety: a survey of physicians in teaching hospitals. Arch Intern Med. (2008) 168:40–6. 10.1001/archinternmed.2007.12 18195194

[6] Seys D Scott S Wu A Van Gerven E Vleugels A Euwema M. Supporting involved health care professionals (second victims) following an adverse health event: a literature review. Int J Nurs Stud. (2013) 50:678–87. 10.1016/j.ijnurstu.2012.07.006 22841561

[7] Wu AW. Medical error: the second victim. The doctor who makes the mistake needs help too. BMJ. (2000) 320:726–7. 10.1136/bmj.320.7237.726 10720336 PMC1117748

[8] Scott SD Hirschinger LE Cox KR McCoig M Brandt J Hall LW. The natural history of recovery for the healthcare provider “second victim” after adverse patient events. Qual Saf Health Care. (2009) 18:325–30. 10.1136/qshc.2009.032870 19812092

[9] Sirriyeh R Lawton R Gardner P Armitage G. Coping with medical error: a systematic review of papers to assess the effects of involvement in medical errors on healthcare professionals' psychological well-being. Qual Saf Health Care. (2010) 19:e43. 10.1136/qshc.2009.035253 20513788

[10] Menon Nikitha K. Association of physician burnout with suicidal ideation and medical errors. JAMA Netw Open. (2020) 3.12:e2028780–e2028780. 10.1001/jamanetworkopen.2020.28780 33295977 PMC7726631

[11] Brown B. Daring Greatly: How the Courage to Be Vulnerable Transforms the Way We Live, Parent, and Lead. New York, NY: Penguin Group. (2012).

[12] Bynum WE Goodie JL. Shame, guilt, and the medical learner: ignored connections and why we should care. Med Educ. (2014) 48:1045–54. 10.1111/medu.12521 25307632

[13] Engel KG Rosenthal M Sutcliffe KM. Residents' responses to medical error: coping, learning, and change. Acad Med. (2006) 81:86–93. 10.1097/00001888-200601000-00021 16377827

[14] Roberts KH. Some characteristics of one type of high reliability organization. Organ Sci. (1990) 1:160–76. 10.1287/orsc.1.2.16034754208

[15] Christianson MK Sutcliffe KM Miller MA Iwashyna TJ. Becoming a high reliability organization. Crit Care. (2011) 15:314. 10.1186/cc10360 22188677 PMC3388695

[16] Pratt S Kenney L Scott SD Wu AW. How to develop a second victim support program: a toolkit for health care organizations. J Commun J Qual Patient Saf . (2012) 38:235–40, 193. 10.1016/S1553-7250(12)38030-6 22649864

[17] Moran D. Cost-benefit analysis of a support program for nursing staff. J Patient Saf 16.4 (2020):e250–e254. 10.1097/PTS.0000000000000376 28452914

[18] Plews-Ogan M. Wisdom in medicine: what helps physicians after a medical error? Acad Med. (2016) 91:233–41. 10.1097/ACM.0000000000000886 26352764

[19] Ignatius E Kokkonen M. Factors contributing to verbal self-disclosure. Nord Psychol. (2007) 59:362–91. 10.1027/1901-2276.59.4.362

[20] Sorokin R Claves JL Kane GC Gottlieb JE. The near miss resident conference: understanding the barriers to confronting medical errors. Semin Pract. (2002) 5:17.

[21] Halbach JL Sullivan LL. Teaching medical students about medical errors and patient safety: evaluation of a required curriculum. Acad Med. 80.6 (2005): 600–06. 10.1097/00001888-200506000-00016 15917366

[22] Provera B Montefusco A Canato AA. “No blame” approach to organizational learning. Br J Manag. (2010) 21:1057–74. 10.1111/j.1467-8551.2008.00599.x

[23] Omarzu J A. disclosure decision model: determining how and when individuals will self-disclose. Pers Soc Psychol Rev. (2000) 4:174–85. 10.1207/S15327957PSPR0402_05

[24] Quality Interagency Coordination Task Force. Doing what counts for patient safety: federal actions to reduce medical errors and their impact. Washington, DC: Quality Interagency Coordination Task Force (QuIC) to the President (2000).

[25] Braun V Clarke V. “Reflecting on reflexive thematic analysis. Qual Res Sport Exerc Health. (2019) 11 589–97.” (2019). 10.1080/2159676X.2019.1628806

[26] O'Brien BC Harris IB Beckman TJ Reed DA Cook DA. Standards for reporting qualitative research: a synthesis of recommendations. Acad Med. (2014) 89:1245–51. 10.1097/ACM.0000000000000388 24979285

[27] West R Turner L. Introducing Communication Theory; Analysis and Application (4th Ed). New York, NY: McGraw-Hill International Edition. (2010).

[28] Jourard SM. Self-Disclosure: An Experimental Analysis of the Transparent Self. Oxford: John Wiley (1971).

[29] Ullström S Sachs MA Hansson J Ovretveit J Brommls M. Suffering in silence: a qualitative study of second victims of adverse events. BMJ Qual Saf. (2014) 23:325–31. 10.1136/bmjqs-2013-002035 24239992 PMC3963543

[30] West CP Huschka MM Novotny PJ Sloan JA Kolars JC Habermann TM. Association of perceived medical errors with resident distress and empathy: a prospective longitudinal study. JAMA. (2006) 296:1071–8. 10.1001/jama.296.9.1071 16954486

[31] Kroll L Singleton A Collier J Rees Jones I. Learning not to take it seriously: junior doctors' accounts of error. Med Educ. (2008) 42:982–90. 10.1111/j.1365-2923.2008.03151.x 18823517

[32] Gray S. Fail better with a failure friend. FEMINEM (2017). Available from: https://feminem.org/2017/12/05/fail-better-failure-friend/ (retrieved on March 21, 2022)

[33] Hobgood C Hevia A Tamayo-Sarver JH Weiner B Riviello R. The influence of the causes and contexts of medical errors on emergency medicine residents' responses to their errors: an exploration. Acad Med. (2005) 80:758–64. 10.1097/00001888-200508000-00012 16043533

[34] Miller LC Berg JH Archer RL. Openers: individuals who elicit intimate self-disclosure. J Pers Soc Psychol. (1983) 44:1234–44. 10.1037/0022-3514.44.6.1234

